# Current knowledge on the mechanism of atherosclerosis and pro-atherosclerotic properties of oxysterols

**DOI:** 10.1186/s12944-017-0579-2

**Published:** 2017-10-02

**Authors:** Adam Zmysłowski, Arkadiusz Szterk

**Affiliations:** National Medicines Institute, Department of Spectrometric Methods, 30/34 Chełmska,, 00–725 Warsaw, Poland

**Keywords:** Cholesterol, Atherosclerosis, Oxysterols, Cholesterol oxidation, LDL

## Abstract

Due to the fact that one of the main causes of worldwide deaths are directly related to atherosclerosis, scientists are constantly looking for atherosclerotic factors, in an attempt to reduce prevalence of this disease. The most important known pro-atherosclerotic factors include: elevated levels of LDL, low HDL levels, obesity and overweight, diabetes, family history of coronary heart disease and cigarette smoking. Since finding oxidized forms of cholesterol – oxysterols – in lesion in the arteries, it has also been presumed they possess pro-atherosclerotic properties. The formation of oxysterols in the atherosclerosis lesions, as a result of LDL oxidation due to the inflammatory response of cells to mechanical stress, is confirmed. However, it is still unknown, what exactly oxysterols cause in connection with atherosclerosis, after gaining entry to the human body e.g., with food containing high amounts of cholesterol, after being heated. The in vivo studies should provide data to finally prove or disprove the thesis regarding the pro-atherosclerotic prosperities of oxysterols, yet despite dozens of available in vivo research some studies confirm such properties, other disprove them. In this article we present the current knowledge about the mechanism of formation of atherosclerotic lesions and we summarize available data on in vivo studies, which investigated whether oxysterols have properties to cause the formation and accelerate the progress of the disease. Additionally we will try to discuss why such different results were obtained in all in vivo studies.

## Background

Despite a significant focus of many researchers on atherosclerosis in humans, the mechanisms of this disease are still not fully understood. Complications occurring in direct connection with atherosclerosis represent a leading global cause of death and disability [1]. Numerous studies have shown that elevated serum low-density lipoprotein (LDL) with sedentary habits is the crucial factor for the initiation and progression of atherosclerosis [2]. However, it is important to define other risk factors, which could influence both the development and progression of atherosclerosis, beyond genetic factors and high LDL cholesterol. Based on cellular and molecular interactions in the formation of atherosclerotic lesions, researchers suspect several pro-atherosclerotic factors. One recently featured is a high concentration of oxidized forms of cholesterols – oxysterols – in the blood [3–9]. Additionally, it has been proven that oxysterols can be formed from cholesterol in food, during storage or cooking. Elevated temperature, which is almost always used to prepare meals, accelerate the oxidation process of cholesterol. For this reason oxysterols from the dietary sources, introduced to human body and to the blood stream, can play a key role of progression of atherosclerosis. Yet, despite increasing in vivo studies, obtained results are divergent, thus the final conclusion about the pro-atherosclerotic properties of oxysterols cannot be drawn. In this review we present current knowledge about the mechanism of atherosclerosis, which tries to presenting, which part of this disease oxysterols are involved. Furthermore, we present data from studies suggesting pro- or anti-atherosclerotic properties and discuss why the differences between the received results have occurred.

## Current knowledge on the mechanism of atherosclerosis

Before we describe the pro-atherosclerotic properties of oxysterols in detail, we have to highlight current knowledge on the mechanism of atherosclerosis. It seems to be the essential aspects to explain the sterols and oxysterols role in development of this civilisation disease.

### The role of endothelium cells. Laminar flow and its anti-atherosclerotic properties

Atherosclerosis is a disease that affects the multifocal repetitive regions of the arterial tree. The formation of this disease begins in the most vulnerable sites (near the branch points and along the inner curvature or regions in which the uniformity of the blood flow is somehow disturbed [10]) by the abdominal aorta, coronary arteries and iliofemoral arteries [11]. Impaired laminar flow induces a small and oscillating shear stress on the artery wall, where endothelial cells (EC) may react by different mechanosensors (PCAM-1/VE-cadherin/VEGFR2) to this mechanical stress [12]. An endothelial cell responses to shear stress by increased synthesis of vasoactive mediator - nitric oxide (NO) to control vascular tone, which causes an immediate reduction in shear stress. Additionally, the cell reacts by secrete extracellular matrix proteins and matrix metalloproteinases to promote remodelling and repair, and with expression of growth factors, e.g. TGF-β, to control cell survival and proliferation [13]. However, if the shear stress is still present, through mechanotransduction, the NF-κB path is triggered as part of the inflammatory process. Elevated NF-κB activity results in the expression of NF-κB-dependent genes, which encode adhesion molecules, such as PCAM-1, ICAM-1, VCAM-1, P-selectin and E-selectin, cytokines: TNFα, IL-1, IL-6, IL-12 and growth factors G-CSF, M-CSF [12, 14]. The importance of the NF-κB pathway in the formation of an atherosclerotic lesion is shown in a study on apoE-knockout mice, in which the applied endothelial-targeted inhibition of NF-κB signalling results in a decrease in the lesion area, which was associated with a decrease in the recruitment of macrophages to the lesion [15]. However, no correlation between decreased lesion area with macrophage NF-kB suppression was found in the study conducted by Kanters et al. (2003) [16].

**Fig. 1:**
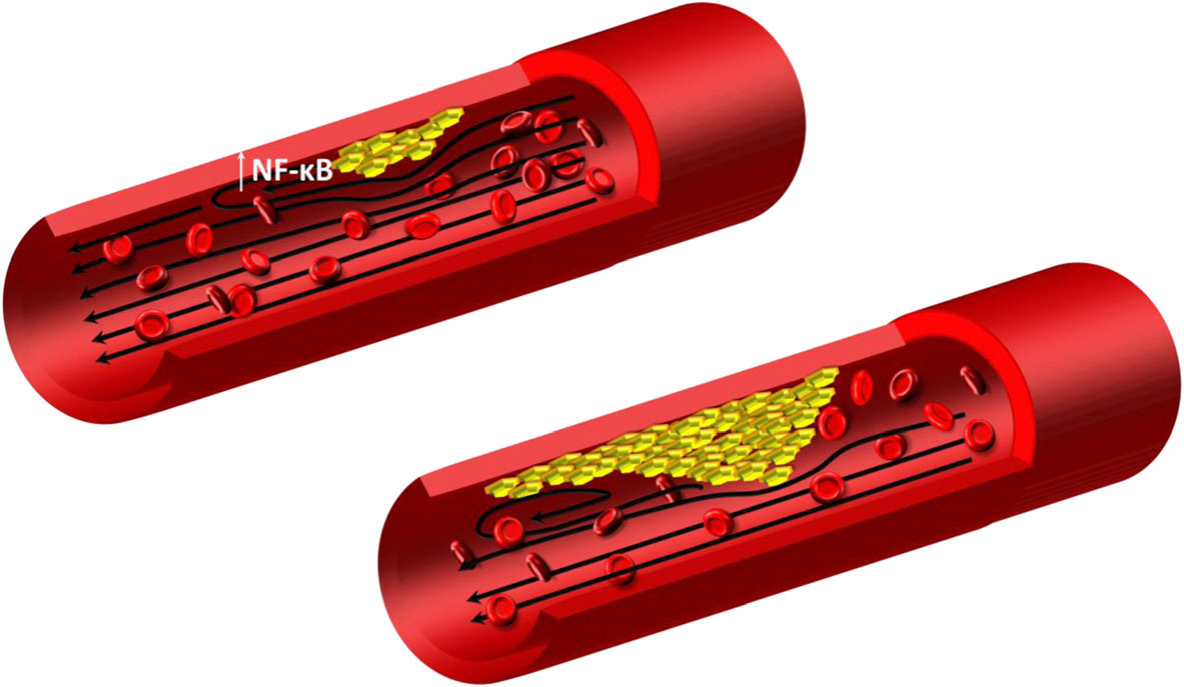
Laminar flow disturbances. Once formed, lesion, leads to maintaining flow disturbances and activation of NF-κB in subsequent cells

The discovery and importance of the activation of this nuclear factor gave an idea that atherosclerosis is not a disease strictly connected with the aging process, but rather a chronic inflammatory condition, which after evolving could lead to intimal destruction, arterial thrombosis and end-organ ischemia. Regardless of mechanotrandsuction, turbulent flow also disturbs intercellular tight junctions of the endolethium and causes thinning of the glycocalyx, which could favour migration of low-density lipoprotein (LDL) and white blood cells to the subendothelial intima [17]. Entry of LDL and diapedesis of monocyte and lymphocyte type T into the intima, begins a cascade leading to the formation of a atherosclerosis lesion. Once formed, the atherosclerotic plaque on the artery wall maintains disturbances in the laminar blood flow, which leads to mechanotransduction and inflammatory activation of nuclear factor NF-κB in subsequent cells of the endothelium. This mechanism can explain why grown plaque will also build up in time along the vessel (Fig. 1) [11].

### The role of LDL and its oxidation in atherosclerosis

A low density lipoprotein (LDL), which is the main carrier of cholesterol in the human body, plays a key role in the transfer and metabolism of this sterol lipid. Due to the fact that lipoproteins are very heterogeneous groups of particles, concerning size and structure, based on physical and physiological properties, it was defined that LDLs are particles having a density of 1.019–1.063 g/ml. More specifically, LDL particles have an average diameter of 22 nm, the core is composed of about 170 triglyceride (TG) and 1600 cholesterol esters (CE) of the molecule and a single surface layer containing approximately 700 molecules of phospholipids (~64% phosphatidylcholine (PC), ~11% lysphosphatdylydcholine (LPC), ~26% sphingomieline) and one copy of the apoB-100 [18, 19].

After mechanotransduction of the endolethium cells, the LDL penetration to intima is possible. Propound mechanism of LDL penetrating into intima are transcytosis, by vesicular bodies across the endothelial cell [20, 21] and sieving through porous pathways between or through the endothelial cells [22]. Mathematical simulation of the atherosclerosis formation supports the idea that passage of LDL is done by sieving via pores through or between endothelial cells [23, 24]. Losing recognition by LDL receptor by its methylation, did not affect penetrability of the particle though the arterial wall in rabbits, which suggests that transfer of the LDL into intima is not LDL receptor dependent [21, 25]. In addition, LDL penetrates the arterial wall as intact particles, however surface free cholesterol and phospholipids are probably exchanged between endothelial cells and the LDL particle [26]. After penetrating the arterial wall, particles bind with intimal proteoglycans [27], which may enhance the susceptibility of the lipoprotein to oxidation [28].

**Fig. 2:**
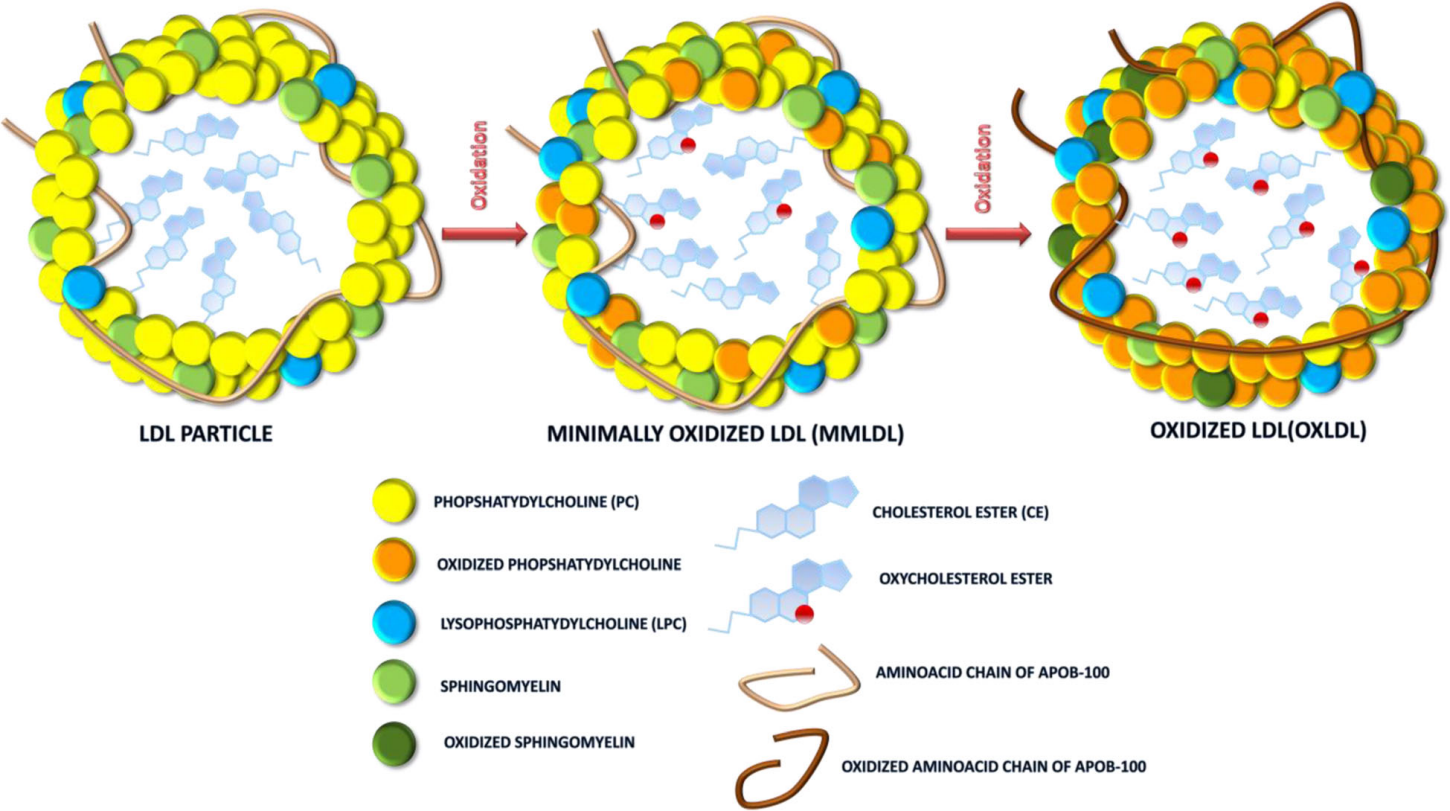
Possible transformation of a LDL particle due to a reaction with highly reactive oxygen species

After gaining entrance to the intima, LDL undergoes various modifications including oxidation, acylation, lipolysis, proteolysis, aggregation and fusion [29–31]. The main reaction occurring is oxidation, primarily by reactive oxygen species (ROS). Early on, oxidation of the LDL leads to the formation of a minimally oxidized LDL (mmLDL), which demonstrates pro-inflammatory activity. Further oxidation of the mmLDL progresses to form moderately oxidized LDL (moLDL) and finally aggregates of highly oxidized LDL (oxLDL) (Fig. 2). MmLDL, moLDL and oxLDL could be recognized by macrophage scavenger receptors (TLR-4, SR-A1,2, CD36 (SR-B2), CD68(SR-D1), LOX-1(OLR1, SR-E1), FcγRII-R2) [32, 33] and each receptor recognises oxidized LDL in a different state: TLR-4 recognizing oxidized cholesteryl esters of mmLDL; LOX1 and SR-A1,2 recognizes modification on apoB-100 of moLDL and oxLDL; CD36 receptor recognizes oxidized phospholipids of moLDL and oxLDL [34, 35]. According to Stanton et al. (1992) [36] high affinity was found in FcγRII-R2 to oxLDL, yet no possible mechanism of its recognition was proposed [36].

Modifications of LDL due to oxidation have a dramatic effect on local vascular homeostasis and endothelial cell cytotoxicity [37], which incite an inflammatory response characterized by the chemokine secretion (CCL2, CCL5), an even higher expression of adhesion molecules by the overlying endothelial cells and the stimulation of macrophages and smooth muscle cells to migration and mitogenesis [38]. The modifications also contribute to lipoprotein aggregation and further promote lipoprotein retention. Additionally, oxLDL have been shown to increase the activity of S-adenosylmethionine-dependent methyltransferases, which lead to increased asymmetric dimethylarginine (ADMA), an endogenous inhibitor of endothelium nitric oxide synthase (eNOS) [39]. The enzyme eNOS is responsible for NO biosynthesis, which, as it was mentioned, is responsible for control vascular tone. NO also has inhibitory properties of inflammation by diminishing endothelial permeability [40], VSMC proliferation and migration [41], platelet aggregation and adhesion [42], and leukocyte adhesion [43].

The main source of ROS is probably 12/15-lipoxygenase, given that mice with a disruption of this enzyme had substantially reduced atherosclerosis [44]. Such studies also conducted by George et al., (2001) [45] demonstrated similar results – the knockout of enzyme production resulted in diminishing LDL oxidation and by this, atherosclerosis development. Alternate modifications are possible by enzymes – secretory sphingomyelinase (SMase), secretory phospholipase 2 (sPLA2) and myeloperoxidase (MPO) [46–48]. Sphingomyelinase may also initiate lipoprotein aggregation, cause increased retention and intensify uptake by macrophages [46].

Given that the majority of compounds (PC and CE), included in a LDL particle, mostly consist of at least one unsaturated fatty acid [49], after the ROS have been generated, the chain reaction leading to the formation of CE hydroperoxide and PC hydroperoxide begins. Since the most common unsaturated fatty acid in the LDL is linoleic acid (C18:2) [50], the main products of oxidations are C18:2 hydroperoxide, C18:2 hydroxide, and ketone [51]. LDL contains also oleic, arachidonic, palmitoleic, docosahexaenoic acids [18], so possible oxidation products from those unsatured acids can also occur. Further oxidation can result in the truncation of sn-2 acyl chain, forming short-chain aldehyde or carboxy derivatives. The aldehydes may form adducts with the lysine residues of apoB-100. The 4-hydroxynonenal, a product of truncation of linoleic acid, is one of the most abundant aldehydes in oxidized LDL, which could react with thiols and free amino groups of apoB-100 and cellular proteins [35]. Additionally, formation of one chain of oxidized PC due to hydrophobic interaction between carbon chains could cause it to flip into exterior side of the LDL particle, which may lead to its recognition by macrophage scavenger receptor CD36 [52].

In addition to the oxidation of unsaturated fatty acids, the oxidation of the sterol moiety of cholesterol via the formation of cholesterol peroxides (e.g. 7-hydroperoxycholesterol (7-hpCh), 24-hydroperoxycholesterol) occurs, of which 7-hpCh have the most cytotoxic properties [53], however, it is rapidly decomposed into 3 relative compounds: 7α-hydroxycholesterol (7α-hCh), 7β-hydroxycholesterol (7β-hCh) and 7-ketocholesterol (7-kCh), which may be found in relatively high concentration in foam cells and fatty streaks [54].

Besides PC, cholesterol and its esters each LDL particle contains one copy of apoB-100 particle. During the oxidation of the LDL, the reaction also occurs on the apoB-100, because it has in its structure amino acids susceptible to being oxidized. ApoB-100 consists of 4536 amino acid residues, which makes it one of the largest monomeric proteins known [55, 56]. Each VLDL particle contains single molecule of apoB-100, which could be modified and transformed, after the metabolic process in the circulation, into LDL particle. Therefore the lipoprotein apoB-100 needs to be able to adapt to the changes occurring in structure and composition, which take place in the particle; for example, the diameter of a VLDL particle is reduced by approximately 4–10 times after converting to LDL particle. ApoB-100 also has a particular role in maintaining the structural integrity and controlling the interactions of LDL particles [57].

The impact of the oxidation on the oxLDL structure is understood poorly. There is data showing changes arising during the oxidation reaction using free radicals or reactive oxygen species. Studies using LDL particles and usage of copper ions to propagate oxidation show that the main oxidation on the apoB-100 occurs on the tryptophan and histidine amino acids [58]. This process leads to the formation of oxyhistidine and kynurenine, after the tryptophan conversion. According to Obama et al. (2007) [58] no oxidation occurred on LDL receptor domains in their studies. Using different hydroxyl radical concentrations, Chakraborty et al. (2010) [59], results in the oxidation of Phe, Tyr, Trp, His, Pro and Lys. In the LDL receptor domains (3130–3160 and 3259–3267) [60], Pro3262 oxidation was observed at low peroxide exposure. The results present that even in early stages of LDL oxidation, modifications in secondary structure and physical state of the particle may not be present, although structure of apoB-100 could undergo changes, mainly in tertiary structure, which could be associated with alteration of particle surface [61]. Extensive LDL exposure to reactive oxygen species will cause oxidation of amino acids eventually leading to the modification of the second structure of the protein (primarily in the α-helical and then in the β-structure), which causes the loss of the ability to be recognized by LDL receptors. After significant losses of the structure of apoB-100, the recognition of the particle is taken by the scavenger receptors LOX1 and SR-A1,2 in macrophages [35].

### The role of the macrophage derivative from monocytes

As mentioned above, activation of the NF-κB pathway in endothelium cells leads to an increased production of adhesion molecules VCAM-1, PCAM-1, ICAM-1, P-selectin and E-selectin, which promote the adhesion of monocytes and lymphocytes type T on the surface of the endothelium. Additionally, activated endothelial cells secrete chemoattractants (MCP-1 (CCL2), CCL5) that interact with cognate chemokine receptors on monocytes and promote directional migration to the endothelium [62]. In vivo studies using mice susceptible to atherosclerosis, with genetic deficient in MCP-1 (CCL2) or its receptor, CCR2, have demonstrated significant protection against lesion formation, possibly by a decreased subendothelial monocyte accumulation [63]. After adhesion, leukocytes exploit loosened intercellular junctions and migrate into the intima. Endothelial cells, sensing the presence of the oxLDL, secrete the monocyte chemoattractant protein (MCP-1 (CCL2)) [64, 65], which triggers recruitment of monocytes into the intima. Generated mmLDLs after LDL oxidation stimulate the endolethium to produce a macrophage colony-stimulating factor (M-CSF), which causes monocytes to proliferate and differentiate to macrophages. After the macrophages are present, using the scavenger receptors, they rapidly uptake the oxLDL (or after different modification) by phatocytosis. As it was mentioned, the uptake of oxidized LDL probably occurs via the scavenger receptors, especially of the scavenger receptor (SR-A1,2) and type-B family member, CD36 and CD68 [33, 66]. However, studies on apolipoprotein E (ApoE-deficient mice) with deficient gene-targeting indicate that additional mechanisms of foam cell formation are also present in atherosclerosis [67].

Once ingested, the cholesteryl esters of the LDL are hydrolysed in late endosomes to cholesterol and fatty acids [68]. Free cholesterol released from lysosomes and from rehydrolysed cholesteryl ester can also be transported to the plasma membrane and thus be available for efflux out of the cell [69]. Cholesterol efflux is one of major processes involved in plaque regression when hypercholesterolemia is reversed. The free cholesterol can also undergo reesterification to cholesteryl fatty acid esters (the “foam” of foam cells) catalysed by the ER enzyme acyl-CoA:cholesterol ester transferase (ACAT) [70]. Studies show that oxysterols can also undergo reesterification by ACAT. Moreover, the data suggested that one of the oxysterols, 7-kCh, may be more suitable to activate the site of the enzyme than cholesterol [71]. On the other hand, the study conducted by Maor and Aviram (1994) [72], presents opposite results. After the uptake of the oxLDL by the J774 cells, a very small concentration of 7-kCh esters was measured and based on that, it was concluded that this oxysterol is not a good substrate for the ACAT enzyme. However, the possible explanation of the received low concentration of the oxysterol ester was limited access to fatty acids available for the esterification reaction [73]. The reason of that was that most of the fatty acids were modified during the copper induced oxidation [74]. Surprisingly, mice ACAT-deficient are still able to develop significant lesions, which suggests that the formation of foam cells is more complex [75].

### The role of the vascular smooth muscles cells and progression of the atherosclerosis lesions

Vascular smooth muscle cells (VSMCs) are a highly specialized cells, whose primary function is to adjust the diameter of the blood vessels and local blood pressure, thereby controlling blood flows in vessels. VSMCs exhibit phenotypic and functional plasticity, mainly by expressing a unique ‘SMC markers’, including: α-smooth muscle actin, smooth muscle-myosin heavy chain, smoothelin- A/B, SMemb/non-muscle MHC isoform-B and cellular retinol binding protein [76]. In case of the vessel damage, VSMCs are able to switch from “normal-contractile” to the ‘proinflammatory-synthethic’ phenotype [77]. This adjustment results in reduction in the expression of mentioned markers responsible for VSMCs contraction and in the production of proinflammatory mediators, which trigger proliferation and migration, a main processes in the vascular wall repair. Moreover cytokines, shear stress, reactive oxygen species, and accumulated lipids, which are present in formation of atherosclerotic plague, can not only altered VSMCs to proinflamatory phytype, but causing abnormal regulation, which leads to additional VSMC dedifferentiation and increased extracellular matrix formation, such as collagen, elastin and proteoglycans [78]. Present oxidized phospholipids from oxLDL inhibit the Krüppel-like factor 4 causes VSMCs to exhibit smooth muscle-derived cells macrophage’ phenotype [79]. However, gene expression of these VSMC-derived macrophage-like cells is definitely different from classical monocytes and macrophages, resulted for example in reduced phagocytic capacity compared with activated peritoneal macrophages.

All process related to unregulated VMSCs – migration, proliferation in the intima, dedifferentiation and the synthesis of extracellular matrix components contribute to progression of the lesion. Accumulated macrophages and migrated VSMCs often die by apoptosis, causing the entering and aggregation of their interior cellular components in the intercellular matrix, which mainly constitute the lipid or necrotic core of the lesion. Local dynamic forces, including circumferential flexion and shear stresses, are main factors, among various, that may destabilize plaques and promote thrombosis [80]. An important factor, which can cause disturbances in the stability of atherosclerotic lesions, is also the possible calcification and neovascularization, common features of advanced lesions. Intimal calcification is associated with pericyte-like cells secreted matrix components, which are subsequently subjected to calcification. This process, which is similar to bone formation, is regulated by oxysterols and cytokines [81]. Thrombosis, the ultimate complication of atherosclerosis, is associated with the eruption of plaque, which causes blood coagulation components to interact with tissue factors in the plaque’s interior, triggering the thrombus, that could extend into the vessel lumen, where it will interfere or block the blood flow. Furthermore, the thrombus can shear off and block important capillary vessels causing a lack of blood flow and ischemia of the cells or important tissues [82].

## The role of oxysterols in atherosclerosis

**Fig. 3:**
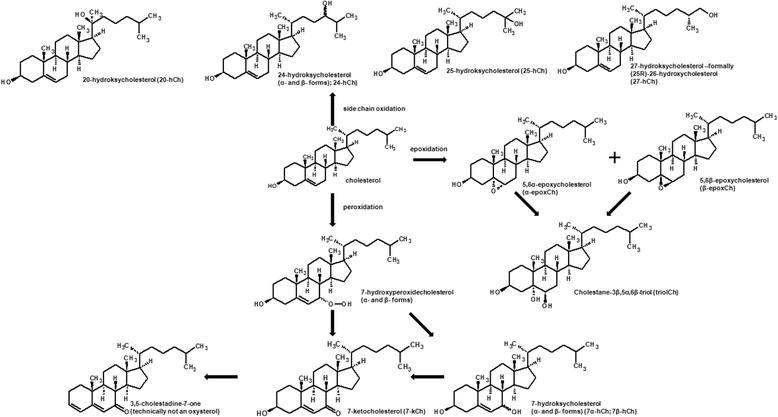
Main modification of cholesterol due to the oxidation occurring in different sites of the molecule [74, 75]

As it was mentioned, oxysterols form during the LDL oxidation, after penetrating into intima. However, there is another possibility, from which oxysterols can be found in human body e.g. in plasma. Cholesterol is very susceptible to auto-oxidation, in a reaction, in which free radicals or highly reactive species participate. Due to the exposure of cholesterol to air during heating or long-term storage, it slowly oxidizes to an epimeric mixture of hydroperoxides, which decomposes to 7-kCh, 7α-hCh, 7β-hCh. Cholesterol-5α,6α-epoxide (α-epoxCh), cholesterol-5β,6β-epoxide (β-epoxCh) are products of epoxidation. The hydration of epoxysterols produces 5α-cholestane-3β,5,6β-triol (triolCh) [83]. There is also a possibility that the side chain oxidizes forming different 20-, 24-, 25- and 27-hydroperoxides and that process often takes place during auto-oxidation of crystalline cholesterol (Fig. 3) [84].

Therefore, oxysterols could be found in various cholesterol-rich foods, even in raw meat, like pork loins, which could be even 0,67% of total cholesterol content [85]. The content of oxysterols can differ much due to different food, method of its preparing, additionally storage and reheating. The highest oxysterol obtained by Min et al. (2016) [85] was triolCh (0,6 mg/100 g pork loins) after pan roasting with additional storage and reheating, which sum up with other oxysterols to about 1,36% of total cholesterol content. The highest oxysterol obtained by Lee et al. (2006) [86], also after pan roasting with additional storage and reheating, was 7-kCh (3,5 mg/100 g beef loins) and taking into account all of the found oxysterols, cholesterol was subject to oxidation in about 6%.

After meal consumption, oxysterols can also be found in human plasma as a result of the absorption of those compounds from intestines [87]. Different absorption rates were achieved by Osada et al. (1994) [88], where only approximately half of the oxysterols was absorbed, comparing to the cholesterol. It was also pointed that absorption rate of individual oxidized cholesterols differed considerably and was approximately 30% for 7α-hCh, 42% for 7β-hCh, 32% for β-epoxCh, 28% for α-epoxCh, 15% for triolCh and 12% for 7-kCh. It was also pointed out, that increased polarity of triolCh was not correlated with its absorption. A lower solubility of oxysterols in bile salt micelles, a lesser susceptibility of oxysterols to esterification in intestinal mucosal cells, and direct toxic effects on mucosal cells were suspected to be reasons for the observed differences [88]. The different absorption rate was also confirmed in a different study conducted by Linseisen and Wolfram (1998) [89], where the highest absorption was obtained by 7α-hCh and 7β-hCh and the lowest with triolCh, which corresponds to results achieved by Osada et al.(1994) [88] However, studies conducted by Krut et al. (1997) and Vine et al. (1997) [90, 91] show that the addition of polar ketone, hydroxide or epoxide moiety to the sterol chemical structure shifts properties of oxysterols to more hydrophilic and by this absorption of these compounds are much more likely than of cholesterol itself. After intestines absorption, oxysterols are mainly transported in plasma bound with albumin or carried by lipoproteins [92]. Oxidized cholesterol are secreted from enterocytes into lymph as incorporated in chylomicrons or chylomicrons remnants at first, yet after chylomicrons regonision by the lipid receptors in hepatocystes, they are passed to VLDL and HLD fraction. VLDL in the circulation is converted via hydrolysis of constituent lipids into smaller LDL particles [87]. So, mainly oxysterols circulate in blood stream in LDL and HDL fraction and in this state they can stay in the human body for a long time [93], which means that the increased polarity does not alter the distribution between different lipoproteins fraction, it is similar to that of cholesterol [94]. Reported oxysterol data in biological samples includes nine oxysterols measured in human plasma, namely: 7-kCh, 7α-hCh, 7β-hCh, α-epoxCh, β-epoxCh, triolCh, 24-hydroxycholesterol (24-hCh), 25-hydroxycholesterol (25-hCh) and 27-hydroxycholesterol (27-hCh) [95]. Additionally, some oxysterols (7α-hC, 24-hCh, 25-hCh, 27-hCh) are biosynthesized in cells by specific enzymes in cytochrome P-450, which are mainly used in the human organism for the conversion into bile acids [96].

Whether oxysterols possess atherogenic properties, their complete removal from the human body is impossible and the question still remains, what oxysterols cause in connection with atherosclerosis, after entering the human body. Cytotoxicity, inducing inflammation, apoptosis and phospholipidosis are confirmed properties of oxysterols on vascular cells [97–99]. Additionally, Sentzu et al. (2012) [100] proved that oxysterols may affect control stiffness in the endothelium cells, after their incorporation into the cell membrane, which may be important in the initiation of the disease. However, due to the fact that atherosclerotic lesions appear in repeating areas, where the laminar flow is somehow disturbed, it suggests that the effect of oxysterols in initial stage of the disease is lower than the presence of the shear stress. Probably oxysterols have much higher effect on already formed atherosclerotic lesions. Because, mainly oxysterols are transported with LDL particle, thus it is possible to diffuse those LDL-oxysterol containing particles into the intima at any point of the formation of atherosclerosis lesion. Based on research conducted by the Vine et al. (1998) [101] LDL containing oxysterols makes lipids more prone to oxidation. Additionally, Staprans et al. (2003) [93] proved that the whole LDL molecule containing oxysterols is more susceptible to reaction with reactive oxygen species. Hence the introduction of the LDL particles may result in oxysterols comprising accelerating the oxidation of LDL to oxLDL and then absorbed by the macrophages. Based on research conducted by Gelissen et al. (1996) [71] the enzyme ACAT catalyzing production of “core” of foam cells can be more likely to esterify oxysterols than cholesterol, and this formation of lesion could be faster when the oxysterol content is higher. In addition, oxysterols may also act at a later stage of the disease. Sato et al. (2012) [102] proved that oxysterols may affect the acceleration of destabilization and rapture of formed lesion. Similar destabilization effects was found by Gargiulo et al. (2016) [103] in apoE^−/−^ mice feed oxysterols, which was prevented when oxysterols absorption was inhibited.

Based on the mechanism of formation of lesion, it seems that oxysterols probably mostly accelerate already formed changes in a blood vessel. The in vitro research and in vivo test on animals should provide data to finally prove or disprove the thesis about the properties of oxysterols. Yet, despite dozens of available publications, results are divergent, thus the study about pro- or not atherosclerotic properties, is still not resolved.

### Data analysis of studies on oxysterols

Prior to in vivo research, essential information of oxysterols properties can be provided using established cell cultures. To investigate whether oxysterols can have an effect on the development of atherosclerosis, study their effect on cells whose involvement in this disease is certain, ie, endothelial cells, smooth muscle cells, macrophages and lymphocytes, should be conducted. Numerous research have shown that oxysterols can significantly induce apoptosis or may cause necrosis on vascular cells [104–106]. Additionally, several cell death mechanisms have been proposed, including: early cytoplasmic membrane modification, cholesterol replacement in membranes by oxysterole associated with disturbances in their essential properties [107], calcium influx [108], overproduction of oxygen radicals [109], loss of transmembrane mitochondrial potential [110], a mitochondrial release of various proteins (cytochrome c, apoptosis-inducing factor, and endonuclease G) into the cytosol [111–114], bcl-2 protein downregulation [115], activation of different caspases (caspase −3, −7, −8 and −9), internucleosomal DNA fragmentation, and condensation and/or fragmentation of the nuclei [112, 114, 116, 117].

Unfortunately majority of available data on effects of oxidized cholesterol on cells are conducted using only single oxysterol. In order to more precisely mimic influence of oxysterols on human vessel cells, after ingestion of oxysterol from dietary sources, in vitro studies should be performed using oxysterol mixtures. Data result from such experiments are available, however inconsistent results were obtained. Larsson et al., (2006) [118] using a mixture of two (concentration 21 μmol/L consisted of 7-kCh (63,9%): 7β-hCh (36,1%)) resulted in induction of necrosis, however mixture of four oxysterols (concentration 21 μmol/L consisted of 33.3% 7-kCh: 7β-hCh (18,9%): 27-hCh (45,7%): 25-hCh (2.0%)) had proapoptotic effect on macrophage cells. In addition, he demonstrated that the use of the four oxysterols exhibit synergistic effect in triggering the apoptosis process. However, Biasi et al., (2004) and Leonarduzzi et al., (2004) [119, 120] usage of a mixture of 7-kCh (35%), 7α-hCh (5%), 7β-hCh (10%), α-epoxCh (20%), β-epoxCh (20%), triolCh (9%), and 25-hCh (1%) resulted in no cytotoxic effects.

The reason for receiving contrary results, in the case of oxysterols mixture, is probably due to its different composition. However already in vitro studies point out that the issue of the influence of oxidized forms of cholesterol in formation and progression of atherosclerosis is extremely complex, even before consideration of available data from in vivo studies. These data from studies, which were carried out in vivo on various animal models, are summarized in Table 1. Such comparison was already done in 1999 by Brown and Jessup [121], however, since this time a few more studies, which tried to investigate atherosclerotic properties, were conducted.

**Table 1:** Summary of studies, which examined oxysterols atherosclerotic properties

.Ref	Animal system	Diet/administration	Oxysterols	Results	Comments
Studies suggesting pro-atherosclerotic properties of oxysterols					
Jacobson et al., (1985) [122]	Juvenile White Carneau pigeons	3 months 1. Control diet – 0,05% cholesterol 2. Diet with 0,05% cholesterol with 0,3% triolCh of cholesterol		Aortic accumulation of calcium in the cholesterol + triol group was 1.16 + 0.35 mg/g, whereas in the cholesterol-fed group it was 0.82 + 0.27 mg/g, an increase of 42% (*P* > 0.02). Coronary artery atherosclerosis, as measured by percent means lumenal stenosis, was 5.23% ± 5.4, in the cholesterol + triol group as compared to 2.80% ± 1.4 in the cholesterol group, an increase of 87% (*P* < 0.01).	No data about the triolCh standard, how it was prepared or what its purity was.
Mahfouz et al., (1997) [123]	New Zealand white male rabbits	11 weeks		The stained sections of the aorta specimen from the rabbits fed with the control diet revealed a normal intima. The stained section of the aorta from the rabbits fed with OC revealed intimal thickening (atherosclerosis). The stained section of the aorta from the rabbits fed with PC showed less intimal thickening than in rabbits fed with OC.	Oxysterol used in study was from US Pharmacopeia cholesterol exposed to air at room temperature for >15 y. Odd composition of oxysterols (presence of 26-hCh, triolCh) compared to other research. Diet with addition of cholesterol also contains small amounts of oxysterols.
		1. an unmodified nonpurified diet (control diet);			
		2. the control diet plus 0.5% cholesterol			
		3. the control diet plus 0.5% cholesterol, which was characterized by a high concentration of cholesterol oxides;	7-kCh (44,4%), 7α-hCh (1,8%), 7β-hCh (6,3%), β-epoxCh (1,7%), 26-hCh (19,9%), triolCh (25,7%)		
Staprans et al., (1998) [126]	New Zealand white rabbit	12 weeks		Serum cholesterol levels increased to a similar extent in both groups, with the majority of cholesterol in the b-VLDL fraction. Moreover, in the serum b-VLDL fraction and liver, there was a significant increase in the oxidized cholesterol levels. Feeding a diet enriched in oxidized cholesterol resulted in a 100% increase in fatty streak lesions in the aorta.	The oxysterols generated by heating cholesterol at 100 °C for 8 h.
		1. 150 g rabbit chow per day with 0,33% cholesterol was added			
		2. 150 g rabbit chow per day with 0,33% cholesterol was added, 5% of total cholesterol was oxidized	Oxysterols (52%): unknowns (48%)		
			Oxysterols:		
			7-kCh (42%), 7α-hCh (7%), 7β-hCh (20%), α-epoxCh (12%), β-epoxCh (16%), 25-hCh (3%)		
Meynier et al., (2002) [127]	Male Golden Syrian hamsters of Charles River’s breeding	3 months		Based on electron microscopy morphology of coronary arteries indicated the development of atherosclerosis in hamster on hyperlipidemia diet with oxysterols	The oxysterols generated by heating cholesterol at 135 °C dissolved in a lipid matrix (lard) under O_2_–CO_2_ (95:5, *v*/v) stream for 48 h. The fatty acids were saponified and the sterol fraction was extracted with dichloromethane and water.
		1. diet containing 25 g corn oil–fish oil (4:1, w/w)/kg (normolipidaemic diet);			
		2. normolipidaemic diet supplemented with 150 g lard, 30 g cholesterol/kg (hyperlipidaemic diet);			
		3. hyperlipidaemic diet, in which 4 g cholesterol/kg was replaced by a mixture of oxysterols	7-kCh (23,5%), 7α-hCh (6,7%), 7β-hCh (18,5%), α-epoxCh (21,1%), β-epoxCh (17,6%), 7-hpCh (3,0%), unknowns (9,6%)		
Staprans et al., (2000) [125]	LDLR^−/−^ and apoE^−/−^ mouse strains with a C57BL/6 J background	28 and 16 weeks		Results demonstrate that oxidized cholesterol in the diet increases fatty streak lesions in aortas of both LDLR^−/−^ and apoE^−/−^ mice. In LDLR^−/−^ mice fed diets that contained oxidized cholesterol, fatty streak lesions increased from 15.93% to 21.00% (32% increase). In apoE^−/−^ mice, the lesion area increased from 15.01% to 20.70% (38% increase)	The oxysterols generated by heating cholesterol at 100 °C for 16 h.
		1. control diet – regular mouse chow supplemented with 1.0% cholesterol			
		2. control diet except that 5% to 10% of the added cholesterol consisted of cholesterol oxidation products (ie, the diet contained 1% cholesterol and 0.05% to 0.10% oxidized cholesterol).	Oxysterols: unknowns about 1:1		
			7-kCh (40–45%), 7α-hCh (7–10%), 7β-hCh (15–20%), α-epoxCh (10–15%), β-epoxCh (15–20%), 25-hCh (traces)		
Plat et al., (2014) [128]	LDLE^+/−^ mice, female	35 weeks		Serum levels of cholesterol, lipoprotein profiles, cholesterol exposure and inflammatory markers at the end of the experiment were comparable between diet groups. The proportion of severe atherosclerotic lesions was significantly higher after oxysterol (41%; *P* = 0,004) than after control diet consumption (26%)	The oxysterols generated by heating cholesterol at 180 °C for 3 h
		1. high-fat diet			
		2. diet with replaced 10% of cholesterol by oxysterols (0,025 g/100 g diet) 0,03 mg oxysterol/g body weight per day	7-kCh (40,3%), 7α-hCh (4,0%), 7β-hCh (13,8%), α-epoxCh (30,3%), β-epoxCh (5,4%), unknowns (6,1%)		
Umetani et al., (2014) [129]	males and females wild type (apoE^+/+^ cyp7b1^+/+^) apoE^−/−^cyp7b1^+/+^ apoE^+/+^cyp7b1^−/−^ apoE^−/−^cyp7b1^−/−^	12 months all genotype groups were fed standard chow	27-hCh was administrated by subcutaneous injection every 2 days (20 mg/kg body weight)	Size and abundance of lesions were larger in apoE^−/−^ and apoE^−/−^cyp7b1^−/−^ than in wild type and cyp7b1^−/−^, in both males and females. Size and abundance of lesions were larger in apoE^−/−^cyp7b1^−/−^ than in apoE^−/−^ in both males and females. ApoE^−/−^ males had greater and more lesions than females.	The 27-hCh was dissolved in 30% (2-hydroxypropyl)-β-cyclodextrin solution, and the same solution served as the control treatment. Impact of 27-hCh on vascular inflammation, activation of NF-κB, monocytes/macrophages and endothelial cells was also evaluated
Studies diminishing pro-atherosclerotic properties of oxysterols					
Higley et al., (1986) [131]	female rabbits (New Zealand White)	11 weeks		Total number of lesions was equivalent to the cholesterol, oxysterols and cholesterol-oxysterols mixture diet groups, the oxysterols and cholesterol-oxysterols diets did not induce the statistically significant degree of severity of the lesions produced by the cholesterol diet.	The oxysterols generated by heating cholesterol at 110 °C for 64 h. The oxidized cholesterol was purified by chromatography, yet some unknowns are present.
		1. Control diet - 2% corn oil			
		2. Diet with 166 mg/kg/day cholesterol			
		3. Diet with 166 mg/kg/day oxysterols	7-kCh (26,0%), 7α-hCh (4,5%), 7β-hCh (5,3%), α-epoxCh (24,0%), β-epoxCh (17,0%), 25-hCh (2,0%) 7-hpCh (18,2%) unknowns (2,9%)		
		4. Diet with 166 mg/kg/day cholesterol and oxysterols mixture	Chol (35%), 7-kCh (8,0%), α-epoxCh (17,0%), 25-hCh (2,0%), 7-hpCh (10,0%) unknowns (20,0%)		
Vine et al., (1998) [101]	Semi-Lop rabbits	2 weeks		Total plasma oxysterols were significantly elevated in both cholesterol supplemented groups. The concentration of aortic total cholesterol in rabbits fed oxidized cholesterol was increased more than 2-fold compared to unsupplemented and purified cholesterol-fed rabbits. Supplementation of the diet with pure cholesterol caused no significant increase in arterial cholesterol concentration compared to the unsupplemented group. After 2 weeks of supplementation with oxysterols or cholesterol, there were no visible lipid lesions on the abdominal or carotid vessels in either group.	The oxysterols generated by heating cholesterol at 135 °C for 4 h Very short (2 weeks) duration of study. The received results are probably correlated with short time of exposure to sterols. Mixture of oxysterols contains cholest-4-ene-3-one and didn’t contain 7α-hC, which is different from other in vivo studies of oxysterols properties.
		1. standard rabbit chow			
		2. rabbit chow supplemented with 1,0% cholesterol			
		3. rabbit chow supplemented with 1,0% cholesterol auto-oxidized (containing 6,0% oxysterols)	7-kCh (41,8 ± 5,1%) 7β-hCh (17,8 ± 2.2%), α-epoxCh (3,8 ± 0,5%), β-epoxCh (3,8 ± 0,5%), cholest-4-ene-3-one (7,2 ± 1,2%), 25-hCh (4,1 + 0,5%), unknowns (21,3 ± 3,5%)		
Ando et al., (2002) [132]	apoE^−/−^ mice	8 weeks		There was no significant difference in the lesion volume in the aortic valve and the content of cholesterol in the aorta among the mice fed the control, cholesterol and oxysterol diets	The oxysterols generated by heating cholesterol at 150 °C for 12 h. Control diet contains small amounts of *t-butyl* hydroquinone, which acts as antioxidant – not possible to predict the impact of an antioxidant addition into animal diet.
		1. control diet			
		2. control diet containing 0·2 g cholesterol/kg (cholesterol diet)			
		3. control diet containing 0·2 g oxysterols/kg (oxysterol diet).	7-kCh (22,1%), 7α-hCh (5,6%), 7β-hCh (15,2%), α-epoxCh (16,2%), β-epoxCh (15,6%), 25-hCh (2,7%), triolCh (1,3%) unknowns (20,5%)		
Weingärtner et al., (2015) [133]	male apoE^−/−^ mice	4 weeks	7β-hCh was delivered daily by i.p. application	Early atherosclerotic lesion formation was similar between controls (17.2 ± 8.5), i.p. application of cholesterol (14.5 ± 9.1%) and 7β-hCh (7.9 ± 4.5%)	No data found about concentration of cholesterol and 7β-hCh delivered to animals.
		diet - Western type (40 kcal% butterfat, 0,15% w/w cholesterol)			

Jacobson et al., (1985) [122] described the investigation of the properties of triolCh fed to pigeons. Animals were divided into two groups: a control group and group fed a diet containing triolCh for three months. The assumption that triolCh has atherosclerotic properties was drawn from a comparison of calcium accumulation and artery stenosis in both groups. In the group supplemented with oxysterol, the accumulation of calcium and observed narrowing of the aortic lumen was 42% and 87% greater than in the control group, respectively. Unfortunately there is no data available on triolCh – its purity or the synthesis pattern, which should provide data if there was a possibility of additional oxysterols administration to animals.

Further results suggesting the atherosclerotic properties of oxysterols after a test using rabbits as an animal model were submitted by Mahfouz et al., (1997) [123]. The test was performed by dividing animals into 3 experimental groups: a control group, a group with an addition of 0.5% cholesterol and a group with an addition of 0.5% cholesterol containing a high content of oxysterols. After 11 weeks, staining of dissected rabbit aortas was conducted, which revealed that in the groups supplemented with sterols, thickening of the aorta wall was observed, while in the control group such thickening did not occur. In addition, in the group with administered oxidized forms of cholesterol, the thickening of the wall was greater compared to the group with a diet containing pure cholesterol. Furthermore, the composition of the mixture of oxysterols was examined and the presented results deviate much from every composition of oxysterols in similar in vivo studies. The most abundant oxysterols were 7-kCh, 26-hCh and triolCh, which suggests that the main oxidation reactions of cholesterol were peroxidation in carbon C7, C26 and epoxidation of double bond C5-C6. As mentioned above, due to the high temperature, the main reactions of the oxidation of cholesterol are the peroxidation of carbon C7 with a transformation to 7-kCh, 7α- and 7β-hCh, the peroxidation of C25 with a transformation to 25-hCh and the epoxidation of C5-C6 carbon to form α- and β-epoxCh. Therefore the content of the oxysterol mixture received by Mahfouz et al., (1997) [123] has to be related to a very long exposure of cholesterol to the atmosphere (cholesterol standard 15 years old). It should also be noted that such a long exposure to the atmosphere certainly resulted in the formation of other compounds (about 20%), which were also not identified in this study. Additionally, triolCh, whose measured content was about 25% in the oxysterol mixture, probably formed from α- and β-epoxCh in acidic conditions [124]. Ultimately, the final conclusion that arises from the Mahfouz study is difficult to compare against other available in vivo studies, due to different oxysterol content administrated to animals.

Similar studies were carried out by Staprans et al., (2000) [125]. The study was conducted with the use of genetically modified mice: LDLR^−/−^ and apoE^−/−^, which were divided and fed in two distinct groups: a control diet – containing 1.0% cholesterol and a diet contained 5–10% of cholesterol oxidation products. After the end of the study (7 and 4 months for LDLR^−/−^ and apoE^−/−^, respectively) animals in the group supplemented with oxysterols contained over 30% more atherosclerotic lesions. Similar results were obtained by Staprans et al., (1998) [126] in a study conducted with rabbits. The 100% increase in the proportion of atherosclerotic lesions in aortas in the group supplemented with oxysterols was achieved compared to the control group. However, in both studies, oxysterols administered to the animals were produced by heating cholesterol at a temperature above 100 °C, without any purification process of the transformed oxysterols. In those studies conducted by Staprans et al., (1998, 2000) [125, 126] about 50% of the oxysterol mixture were compounds of unknown structure.

The atherogenic properties were also confirmed by Meynier et al. (2002) [127], using hamsters as a test model. Animals were divided into 3 different diet groups: a control diet, a high-fat diet and a high-fat diet containing added oxysterols. After the test (3 months), based on observation using an electron microscope, no significant changes in vessel walls were observed in the control diet and the cholesterol diet. However, in the diet containing oxysterols, the morphology of vessels was so different from a normal morphology, that based on this observation, it was concluded that atherosclerosis was present.

Plat et al., (2014) [128] based on the results of his experiment, suggested that oxysterols increase the number of serious atherosclerotic lesions. The study was performed for 35 weeks with genetically-modified LDL receptor mice, which were divided into two groups: a control group and a group with a similar diet, but 10% of the cholesterol was replaced with a mixture of oxysterols. After the duration of the study cholesterol, lipoprotein cholesterol profile and inflammatory markers were comparable in both groups. However, the number of severe lesions differs by 15% in the group with oxysterols compared to the control group. From the result obtained by Plat, it can be concluded that oxysterols possess pro-atherosclerotic properties, yet generating oxysterols by heating in high temperature casts doubt on this conclusion. Using oxysterols generated by heating without a cleaning process, makes it possible to administrate mice compound of unknown structure and properties. Such compounds were also foundby Plat et al., (2014) [128], which was about 6.1% of all oxysterol mixtures.

Umetani et al. (2014) [129] described study of impact of 27-hCh on atherosclerotic formation. Four different genotypes of mice was use in the study: wild type (apoE^+/+^; cyp7b1^+/+^), apoE^+/+^cyp7b1^−/−^, apoE^−/−^cyp7b1^+/+^ and apoE^−/−^cyp7b1^−/−^. All animals had the same standard chow and the 27-hCh was administrated by subcutaneous injection every 2 days. After 12 months, the analysis of the amount and severity of atherosclerotic plaques was evaluated. Size and abundance of lesions was larger in apoE^−/−^ and apoE^−/−^cyp7b1^−/−^ groups compared to wild type and in cyp7b1^−/−^ group, in both males and females. Additionally, lesions were larger in apoE^−/−^cyp7b1^−/−^ than in apoE^−/−^, in both males and females. Interestingly, apoE^−/−^ males had greater and more lesions than females, which is probably related to protective actions of estrogen [130]. It should be pointed out that such impact of oxysterol on different gender was not tested in others in vivo study. In addition, it was proven that 27-hCh invokes proinflammatory processes in vascular cells both in vitro and in vivo. Macrophage accumulation in the vascular wall was also increased by 27-hCh, proinflammatory genes were upregulated, and plasma TNF-α was elevated. Each result obtained by Umetani study confirmed that increases of the occurrence of aorta atherosclerosis is related to 27-hCh presence in plasma. However conducted study is unique due to usage 27-hCh, an endogenous oxysterol, which could be found in plasma, because it is synthesized in human body by bile enzyme CYP27A1 [96]. Unfortunately it is not possible to compared these result with other studies investigating oxysterols, because they are focused on oxysterols, which do not form in a healthy human body.

In another study Higley et al., (1986) [131] investigated the properties of oxysterols fed to rabbits. Animals were divided into 4 different groups: a control diet, a diet high amount of cholesterol, a diet high amount of oxysterols and a diet high amount of cholesterol and an oxysterol mixture. After 11 weeks, there was no observed difference in the amount and severity of plaque in groups fed with sterols. As in other described studies, oxysterols were produced by heating cholesterol above 100 °C, yet it is worth mentioning that it was the first time that oxysterols were used after a purifying process using preparative chromatography. Unfortunately, although the cholesterol was submitted to a purification process, the content of unknown compounds in the cholesterol/oxysterol mixture was about 20%.

Vine et al., (1998) [101] on the basis of the conducted research using rabbits as an animal model, verified if oxysterols possess atherosclerotic properties. The study was conducted using three groups of animals: having a standard diet, a diet containing 1.0% cholesterol, and having a diet with 1.0% cholesterol with oxysterols. After 2 weeks of supplementation, the levels of oxysterols in the plasma of both groups with a cholesterol diet were significantly elevated. In addition, the cholesterol content in the aortas in the group supplemented with cholesterol and oxysterols was more than twice higher than in the group supplemented with the addition of cholesterol. However, there were no visible atherosclerotic changes in carotid and abdominal aortas in all groups. Based on the received results, Vine et al. concluded that oxysterols do not possess atherosclerotic properties. However, according to the disease mechanism, first the production of lesions and a subsequent progression of oxysterols is needed. For this study such properties cannot be investigated within 2 weeks of the administration of an oxidized form of cholesterol. It is also noticeable that animals were administrated with a mixture formed after cholesterol heating, which contained cholest-4-ene-3-one, which technically is not an oxysterol.

Another study investigating the properties of oxysterols was performed by Ando et al., (2002) [132]. Studies were conducted using apoE^−/−^ mice, which were divided into three groups, each on a different diet – control diet, a diet containing cholesterol and a diet containing oxysterols. After 11 weeks the size of the atherosclerotic lesions in aortas was examined and there was no statistically significant difference between all groups, which leads to the conclusion that oxysterols do not possess atherosclerotic properties. As mentioned in the previously described studies, also in this one, oxysterols were produced by heating cholesterol above 100 °C without any purifying process, which was a reason of obtaining about 20% of unknown compounds in the oxysterol mixture. Furthermore, mice chow contained *t-butyl* hydroquinone, which acts as an antioxidant, which raises a question whether it is possible to protect LDL from oxidation and by this protect mice from lesion formation.

ApoE^−/−^ mice were also used in a study conducted by Weingärtner et al., (2015) [133]. Animals were divided into three groups: a placebo group (dextran emulsion), a group administered with cholesterol and a group administered with 7β-hCh. All groups had a Western-type diet containing 0.15% (*w*/w) cholesterol. After 4 weeks of cholesterol and 7β-hCh by i.p. application, no differences were observed in the number and severity of atherosclerotic lesions in all groups. The study made by Weingärtner et al. (2015) is unique, which makes the obtained result difficult to compare to other available in vivo research. Unfortunately, there is no data in the description concerning the concentration of cholesterol or oxysterol that was administered to the mice. Therefore at this point, it is unclear, if the animals were treated only with pure oxysterol or mixture, again with unknown compounds. Additionally, obtained result cannot be used to draw a conclusion about atherosclerotic properties of other remaining oxysterols, which during heating occur in larger quantities than 7β-hCh. On the other hand, this result could give researchers an idea to study in vivo only one selected oxysterol.

**Fig. 4:**
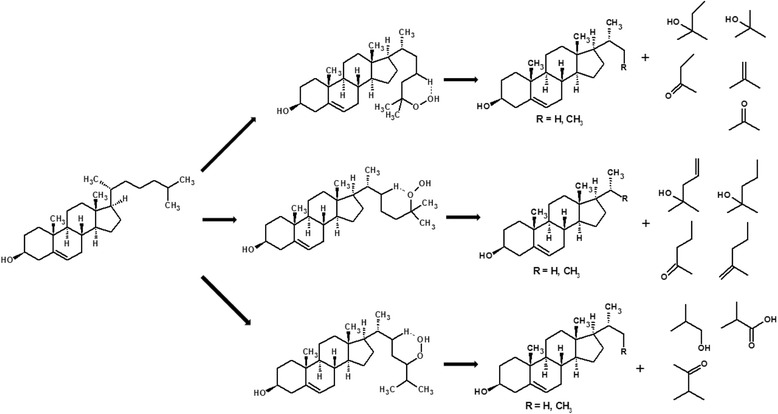
Example of non-typical cholesterol modification due to the peroxidation of the side chain, which causes the formation of volatile compounds and a cholesterol derivative, which could possibly undergo another oxidation

Heating cholesterol to a high temperature, is, for certain, the simplest way of generating oxysterols, however according to available data, analysing the mechanism of cholesterol oxidation, thermal treatment causes the formation of a large number of compounds from cholesterol, including cholestadienes (technically not oxysterols) – which was mentioned only in one study conducted by Vine et al., (1998) [101], fragmented cholesterol (3β,17,21α-pregn-ene, ether of 7α-hydroxypregnenolone and 3β,4β-cholest-5-ene), oligomers (dimmers, trimers), volatile compound molecules (aldehydes, e.g., 3-methyl-butanal, ketones, e.g., 2-pentanone, alcohols, e.g., 3,4-dimethyl-1-pentanol, hydrocarbons, e.g., 2-methyl-4-decene, esters, e.g., hexanoic acid) (Fig. 4) [134, 135].

Due to the fact that in every in vivo study, oxysterols administered to animals were prepared by heating cholesterol at high temperatures, which in fact resulted in the formation of a mixture of oxysterols with compounds of unknown structure and properties, a final and definitive conclusion about pro-atherosclerotic properties of oxysterols should not be drawn. Therefore, the study with animals ought to be carried out after the purifying process of oxysterols to ensure that the animals are fed only with the oxidized forms of cholesterol in a suitable proportion, mimicking their content in food. Only one study conducted by Higley et al., (1986) [131] used a mixture of oxysterols after a purification process, using the preparative HPLC. Even after using chromatography some unknown compounds were still present. Based on presented and analysed data, researches of oxysterols properties in in vivo studies should aim towards a new path – using mixtures of oxysterols after suitable purification process or using oxysterols from synthesis or from commercially available vendors. Not only animals were treated with unknown compounds, but the composition of oxysterols mixture in every study was different. It is possible that not every oxysterol has atherosclerotic properties. Additionally it is also possible that the atherosclerotic effect was found, because of the synergetic effect of oxidized forms [98], which can be various when composition of the mixture also differs. Furthermore time of the administration of oxysterols to animals differed from 2 weeks to 7 months, which is probably due to different animal models. Some animal models are atherosusceptible like some rabbit strains, which exhibit familial hypercholesterolemia or wild type mouse strains, which have much more rapid heart rate hence disturbed laminar blood flow is more possible [136]. However the most frequently used models in study of atherosclerosis are the genetic modified apoE^−/−^ and the LDLR^−/−^ mouse model [137]. Genetic modification in apoE^−/−^ strain resulted in shortened time, in which animals develop atherosclerotic lesions [138]. However, even for the same animal model, experiment time for determination of oxysterols properties, in the two studies conducted by Staprans et al., (2000) [125] and Weingärtner et al., (2015) [133], differed by up to 12 weeks, which may explain obtained opposite results. In addition, not only the choice of animal model is important for studies, but also the choice of the animal diet [136]. Due to the fact that in each of the mentioned in vivo, diet of control group is different, it causes the irregularity of the comparison of these results. Diet chosen for animal control group should be standard or western type chow, which could result in negligence of different times of the oxysterols exposure.

Different model, time of the experiment, different animals chow and the administrated oxysterol/oxysterol mixture in each of in vivo study, are reasons why obtained results should be considered only individually, which makes the final conclusion of atherosclerotic properties of each oxysterols not possible to draw.

## Conclusions/closing remarks

Heart diseases, cellular aging vessel walls can promote turbulence in the laminar blood flow and the formation of atherosclerotic plaques, which is only the final result of a complex and self-perpetuating cell response to this mechanical stress. For this reason, finding factors, which may cause faster progression of already formed changes seems to be appropriate.

Based on the mechanism of atherosclerotic lesion formation and mentioned in vitro and in vivo studies, it is possible that oxidized forms of cholesterol are one of the factors, which accelerate growth that has already been in the vessel wall and have less effect to cause the initiation of the disease. Such hypothesis needs to be tested in vivo after administration of an oxysterol or oxysterols mixtures only after suitable lesion formation, using e.g. Western type diet. Such approach was applied by Plat et al., (2014) [128] and could confirm this assumption; however, as mentioned, the use of high temperature process cholesterol without a purification process, makes the conclusion of the study doubtful.

As it was mentioned, the amount of consumed oxysterols by human could be relatively high (even 6% of total cholesterol content). Therefore, elimination of oxysterols content in a diet may not be possible, but it could be minimized by reducing the consumption of cooked cholesterol-rich food, especially after processed by methods, which use high temperatures. Therefore, it is highly desirable to conduct a study that could ultimately determine whether oxysterols actually possess properties to accelerate the growth of atherosclerosis lesions. If such properties are confirmed, it will cause a significant impact on the patient diet with diagnosed atherosclerosis.

## Abbreviations

24-hCh: 24-hydroxycholesterol
25-hCh: 25-hydroxycholesterol
27-hCh: 27-hydroxycholesterol
7-kCh: 7-ketocholesterol
7α-hCh: 7α-hydroksycholesterol
7β-hCh: 7β-hydroksycholesterol
ACAT: acyl-CoA:cholesterol ester transferase
ADMA: Asymmetric dimethylarginine
apoB: Apolipoprotein B
apoE: Apolipoprotein E
CCL5: Chemokine ligand 5
CE: Cholesterol ester
EC: Endothelial cells
eNOS: Endothelium nitric oxide synthase
G-CSF: Granulocyte-colony stimulating factor
ICAM-1: Intercellular Adhesion Molecule 1
IL: Interleukine
LDL: Low density lipoprotein
MCP-1 (CCL2): Monocyte Chemoattractant Protein 1
M-CSF: Macrophage colony-stimulating factor
mmLDL: Minimally oxidized low density lipoprotein
MPO: Myeloperoxidase
NF-κB: Nuclear factor kappa-light-chain-enhancer of activated B cells
NO: Nitric oxide
oxLDL: Highly oxidized low density lipoprotein
PC: Phosphatidylcholine
PCAM-1: Platelet endothelial cell adhesion molecule 1
ROS: Reactive oxygen species
SMase: Secretory sphingomyelinase
sPLA2: Secretory phospholipase 2
SR-A: Scavenger receptor type A
TG: Triglyceride
TGF-β: Transforming growth factor beta
TNFα: Tumor necrosis factor α
triolCh: 5α-cholestane-3β,5,6β-triol
VCAM-1: Vascular cell adhesion molecule 1
VE-cadherin: Vascular endothelial cadherin
VEGFR2: Vascular endothelial growth factor receptor 2
VLDL: Very low density lipoprotein
VSMC: Vascular Smooth muscle cell
α-epoxCh: Cholesterol-5α,6α-epoxide
β-epoxCh: Cholesterol-5β,6β-epoxide
